# Maternal lipids in overweight and obesity: implications for pregnancy outcomes and offspring’s body composition

**DOI:** 10.1007/s00281-024-01033-6

**Published:** 2025-01-22

**Authors:** Marie Albrecht, Anna Worthmann, Jörg Heeren, Anke Diemert, Petra Clara Arck

**Affiliations:** 1 Department of Obstetrics and Fetal Medicine, University Medical Center Hamburg-Eppendorf, Hamburg, Germany; 2 Junior Research Center for Reproduction: Sexual and Reproductive Health in Overweight and Obesity (SRHOO), University Medical Center Hamburg-Eppendorf, Hamburg, Germany; 3 Hamburg Center for Translational Immunology, University Medical Center Hamburg- Eppendorf, Hamburg, Germany; 4 Department of Biochemistry and Molecular Cell Biology, University Medical Center Hamburg- Eppendorf, Hamburg, Germany

**Keywords:** Lipidomics, Developmental Origin of Health and Disease, Pregnancy complications, Offspring body composition, Overweight and obesity

## Abstract

Overweight and obesity (OWO) are linked to dyslipidemia and low-grade chronic inflammation, which is fueled by lipotoxicity and oxidative stress. In the context of pregnancy, maternal OWO has long been known to negatively impact on pregnancy outcomes and maternal health, as well as to imprint a higher risk for diseases in offspring later in life. Emerging research suggests that individual lipid metabolites, which collectively form the lipidome, may play a causal role in the pathogenesis of OWO-related diseases. This can be applied to the onset of pregnancy complications such as gestational diabetes mellitus (GDM) and hypertensive disorders of pregnancy (HDP), which in fact occur more frequently in women affected by OWO. In this review, we summarize current knowledge on maternal lipid metabolites in pregnancy and highlight associations between the maternal lipidome and the risk to develop GDM, HDP and childhood OWO. Emerging data underpin that dysregulations in maternal triglyceride, phospholipid and polyunsaturated fatty acid (PUFA) metabolism may play a role in modulating the risk for adverse pregnancy outcomes and childhood OWO, but it is yet premature to convert currently available insights into clinical guidelines. Well-designed large-scale lipidomic studies, combined with translational approaches including animal models of obesity, will likely facilitate the recognition of underling pathways of OWO-related pregnancy complications and child’s health outcomes, based on which clinical guidelines and recommendations can be updated.

**Supplementary Information:**

The online version contains supplementary material available at 10.1007/s00281-024-01033-6.

## Introduction

**Fig. 1:**
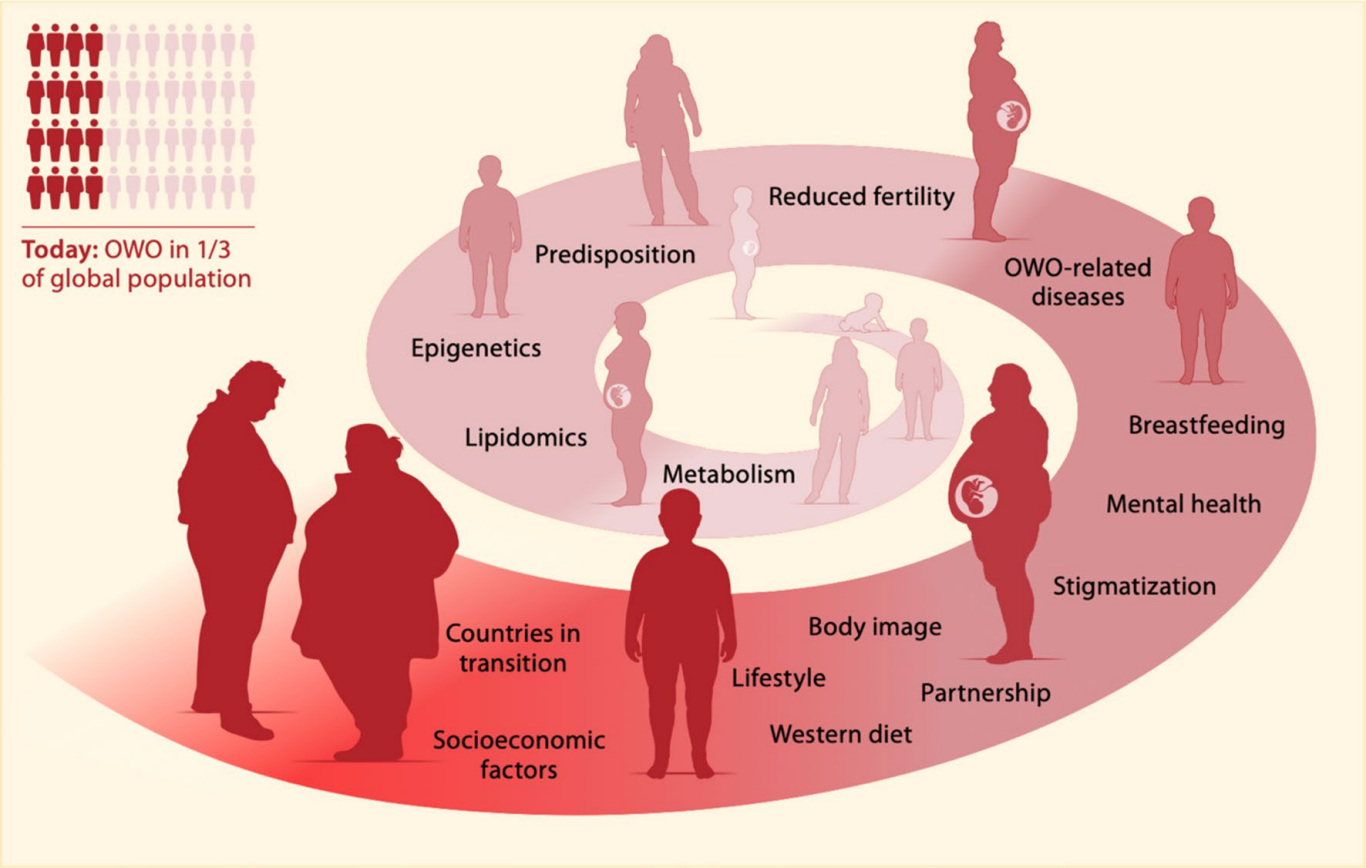
Overweight and obesity (OWO) are global challenges with rising prevalence, multifactorial genesis and consequences that severely impact on reproductive and overall health across many generations. Relevant aspects of OWO etiology, maintaining factors, and consequences are shown

Overweight and obesity (OWO^1^) are steadily increasing worldwide, with emerging prevalence among adolescents and adults during their reproductive years [1]. OWO genesis is multifactorial, and OWO-related pathologies can severely impact on reproductive and overall health across many generations (Fig. 1). Medical conditions that are closely linked to OWO in adults include cardio- and cerebrovascular diseases, metabolic diseases, as well as malignancies [1–3]. Of note, a proportion of individuals with OWO are considered metabolically healthy; this is a transient state that can flip towards metabolic impairment and back, depending on diet and lifestyle [4]. The prevalence of OWO-related diseases distinctively parallels the increase in OWO prevalence, with snow-balling effects on expenses of health care systems [5].

**Fig. 2:**
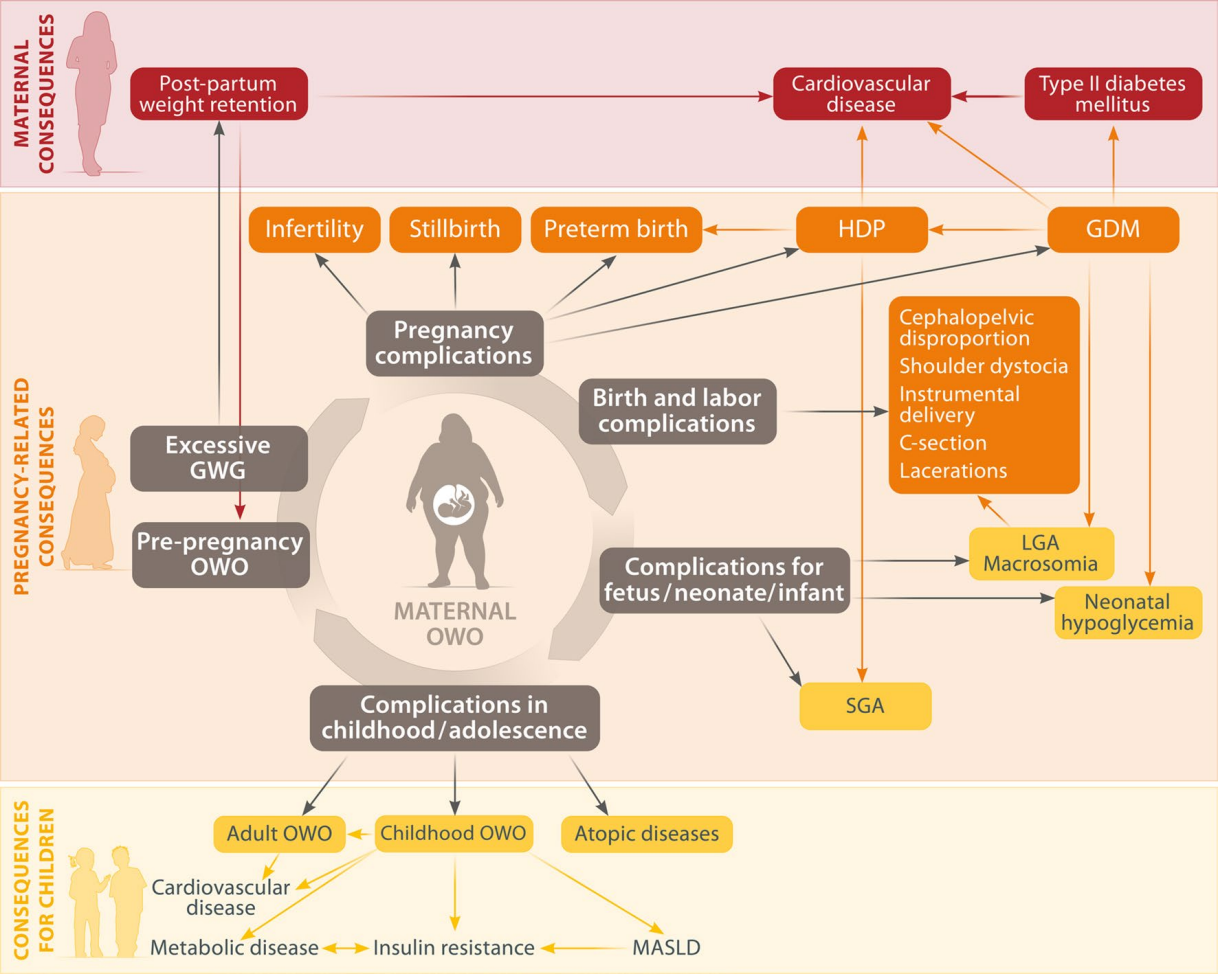
Consequences of maternal pre-pregnancy overweight and obesity (OWO) on pregnancy outcome, maternal and child’s health. Maternal pre-pregnancy OWO increases the risk for excessive gestational weight gain, pregnancy complications, and birth and labor complications. It impacts fetal, neonatal and infant health and has negative long-term consequences for mother and child. Arrows represent potential consequences that have been associated with the indicated condition. C-section: Cesarean section; GDM: gestational diabetes mellitus; GWG: gestational weight gain; HDP: hypertensive disorders of pregnancy, including pre-eclampsia; LGA: large for gestational age; MASLD: metabolic dysfunction-associated steatotic liver disease; OWO: overweight and obesity; SGA: small for gestational age

 Since OWO increasingly affects younger individuals, reproductive health can be jeopardized (reviewed in [6]). In fact, pre-pregnancy OWO is associated with infertility, a higher risk for stillbirth, and pregnancy complications such as gestational diabetes mellitus (GDM), preterm birth, and pre-eclampsia [6, 7] (Fig. 2). Further, complications during labor occur more often in women affected by OWO, along with a higher rate of cesarean sections [6]. Excessive gestational weight gain (GWG), (defined as the exceedance of the IOM reference ranges, which differ for underweight (12.5–18 kg), normal weight (11.5–16 kg) and overweight/obese women (7–11.5 kg/5–9 kg), respectively [8, 9]), occurs more frequently in mothers with pre-pregnancy OWO [10], and has been independently associated with higher risks for GDM, hypertensive disorders of pregnancy (HDP, including pre-eclampsia), birth complications and large for gestational age (LGA) neonates [11–13]. Also, excessive GWG has been demonstrated to increase the risk for post-partum weight retention and long-term disease in the mother, e.g., for type II diabetes mellitus [10, 11]. Hence, interpregnancy weight management is of major importance to avoid entering subsequent pregnancies with retained weight and thus, to avert long-term metabolic consequences in mother and offspring. On the other hand, the failure to gain the minimal recommended amount of weight is considered as an inadequate GWG, which is accompanied by an increased risk for small for gestational age (SGA) neonates [12]. Strikingly, GWG does not appear to be primarily caused by excessive energy intake, but rather by reduced energy expenditure and metabolic adaptation during pregnancy [14].

In line with the Developmental Origin of Health and Disease (DOHaD) notion, OWO and excessive GWG are believed to establish an adverse intrauterine environment and hereby impact fetal development, subsequently leading to negative lifelong health consequences for the child [6, 7, 15, 16]. In fact, maternal pre-pregnancy OWO and excessive GWG have been identified as risk factors for the development of obesity, as well as metabolic and cardiovascular diseases later in the child’s life [6, 7, 17]. Childhood OWO, in turn, negatively affects every organ system and promotes OWO and related diseases in adulthood [18] (Fig. 2).

Although these observations are longstanding, specific mechanisms underlying the vicious circle of OWO perpetuation still remain elusive. Environmental influences *in utero*, such as metabolic changes associated with maternal OWO, seem to largely impact on the programming of fetal development. Several mechanisms have been proposed in this context, including alterations of placental function, epigenetic modifications, increased inflammation, altered mitochondrial function, and others [19, 20]. Along these lines, it is conceivable that maternal OWO-related dyslipidemia may play a role in the development of adverse pregnancy and child’s health outcomes.

**Fig. 3:**
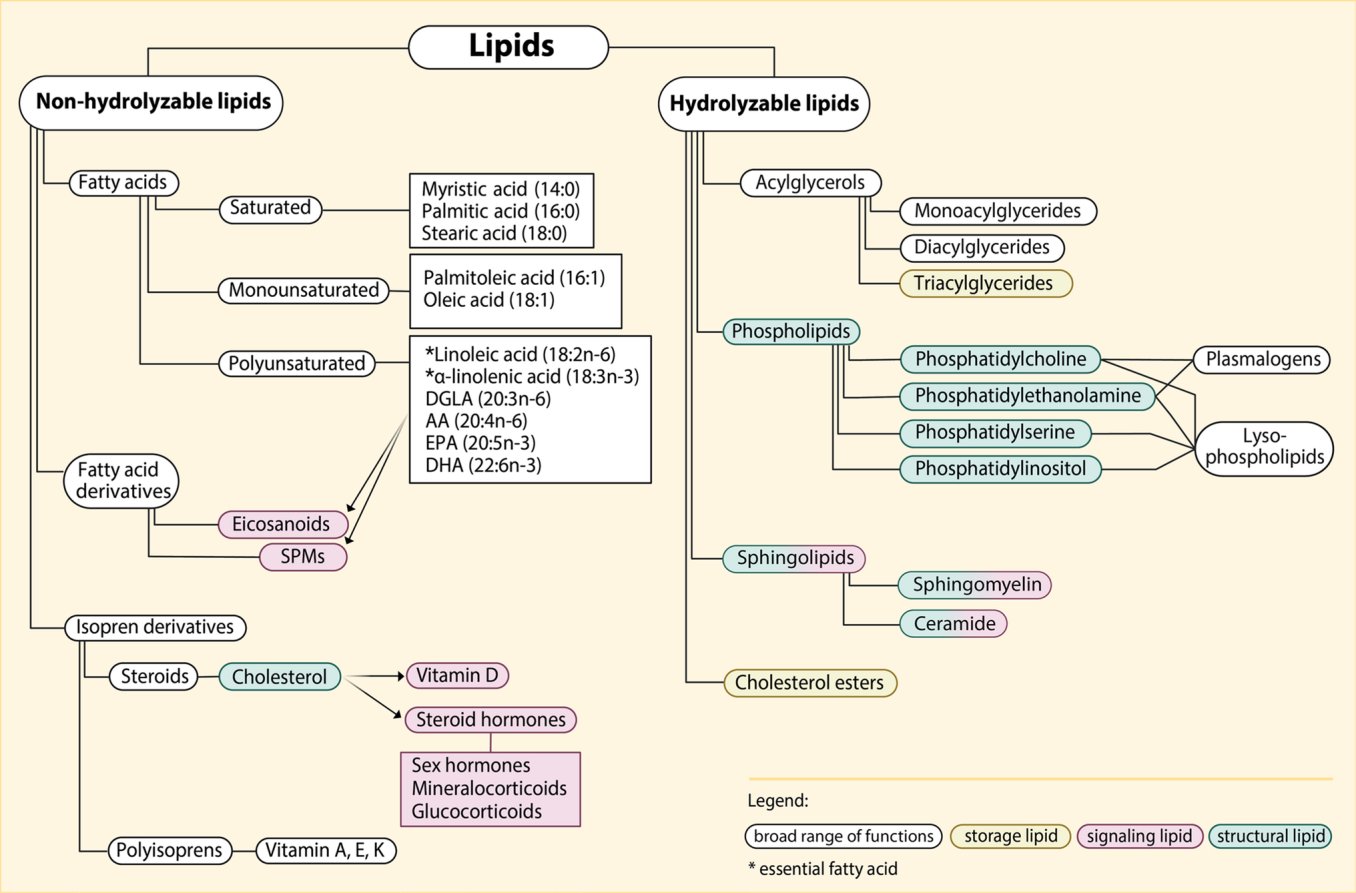
Lipid classification by structure and function. Non-hydrolyzable lipids include fatty acids and their derivatives as well as isopren derivatives. Fatty acids subdivide into saturated, mono- and polyunsaturated fatty acids, depending on the number of carbon double bonds within the molecule. Here, only a small selection of fatty acids is shown. α-linolenic acid (ALA) and linoleic acid (LA) are essential fatty acids that cannot be synthesized by the human body and thus need to be taken up through the diet. Different polyunsaturated fatty acids that derive from α-linolenic acid and linoleic acid serve as precursors for signaling lipids (red), such as eicosanoids and specialized pro-resolving mediators (SPMs). Isopren derivatives include the steroid cholesterol, which serves as a structural lipid (green) in cell membranes, but also as the precursor for vitamin D and steroid hormones. Polyisoprens give rise to several vitamins. Hydrolyzable lipids are more complex, they consist of fatty acids that are bound to an alcohol backbone and various other components. E.g., fatty acids, when bound to a glycerol backbone, form the class of acylglycerols, of which tri(acyl)glycerols are the most abundant storage lipid (yellow) in the body. Phospholipids are the main structural lipids in cell membranes; they consist of fatty acids bound to a glycerol-3-phosphate backbone with either choline, ethanolamine, serine or inositol as their name-giving components. Plasmalogens are similar to phosphatidylcholine and phosphatidylethanolamine, but are characterized by an ether bond instead of an ester bond at the C1 position of glycerol. Sphingolipids include sphingomyelins and ceramides, which both act as structural and signaling lipids. Cholesterol esters are stored in intracellular lipid droplets. Lipids in white boxes have a broader range of functions and thus are not categorized for function in this graphic. The chart was designed on the basis of information provided in references [21–23]. AA: arachidonic acid; DGLA: dihomo-gamma-linoleic acid; DHA: docosahexaenoic acid; EPA: eicosapentaenoic acid; n-3: omega-3; n-6: omega-6; SPMs: specialized pro-resolving mediators

Analysis of blood lipid levels, which are measured in easily accessible venous blood samples, is a promising approach to gain more insight into the respective mechanisms and mediators. However, most studies examining the maternal lipid profile during pregnancy were conducted at times where laboratory methods were not as advanced as today, thus reporting only very broad, standard lipid profiles (e.g., concentration of triglycerides, total cholesterol, and lipoproteins), which can be routinely measured in clinical practice. Although these markers provide a good overview of an individual’s lipid status and metabolic state, they are unable to reflect the abundance of individual lipid species, termed lipidome. As a reflection of their complex functions, lipid molecules are of highly variable structure (Fig. 3), which sometimes only encompass minor changes. Advances in laboratory methods, e.g., modern mass spectrometry or nuclear magnetic resonance spectroscopy, provide the opportunity to study the lipidome with high resolution and to distinguish between lipids with very similar structures. However, lipidome analysis depends on labor-intensive methods which are prone to disruption and require high-level expertise to evaluate and interpret the results, especially in the context of pregnancy. Most pregnancy-related lipidomic studies lack comparability due to varying methodology, study design, choice of participants and blood sampling timepoints, as well as outcomes and metabolites of interest and thus should be interpreted and compared with caution. In addition, most studies only report statistical associations between specific lipids and respective outcomes, which can hint on – but importantly, do not imply – causality in humans.

In this review, we highlight differences in maternal lipids comparing pregnant women with and without OWO, as well as discuss the possible role of individual lipid species in the development of GDM, HDP/pre-eclampsia, and childhood OWO, particularly focusing on maternal OWO as a known risk factor for these conditions. Unless it is indicated otherwise, we herein refer to blood (plasma and serum) lipid analysis in humans.

## Overweight and obesity are accompanied by dyslipidemia

The lipid metabolism is a key regulator in storage and provision of energy and relies on complex pathways. Due to their hydrophobicity, lipids require specific transport vehicles, namely lipoproteins, for their transportation in the blood. Lipoproteins are divided into the main subclasses of chylomicrons, very low density lipoproteins (VLDL), low density lipoproteins (LDL), and high density lipoproteins (HDL). They are characterized by the amount and type of lipids that they carry, as well as by specific apolipoproteins (Apo), which provide structure and are essential for lipoprotein metabolism [24]. In patients with OWO, dyslipidemia is highly prevalent and often follows an atherogenic pattern, which is mainly characterized by hypertriglyceridemia (driven by elevated levels of triglyceride-rich lipoproteins like chylomicrons and VLDL), lower concentrations of HDL-cholesterol (HDL-C) as well as remodeling of LDL with formation of small dense LDL and oxidized LDL [25, 26]. Oxidized LDL, as well as triglyceride-rich lipoproteins and their remnants, contribute to vascular damage by recruiting immune cells, promoting inflammatory cytokine production, and oxidative stress [27]. HDL, on the other hand, is attributed protective properties due to its role in reverse cholesterol transport from the periphery to the liver [28]. Furthermore, HDL cholesterol efflux as well as its anti-inflammatory capacity have been demonstrated to be inversely associated with atherosclerotic cardiovascular disease independently of HDL cholesterol concentration [29, 30].

Another term often used in the context of lipid dysregulation in OWO is *lipotoxicity*. It refers to the adverse effects of lipids that accumulate in non-adipose tissues [31]. A surplus of body fat leads to an exceedance of adipocyte storage capacity, insulin resistance, and increased lipolysis, resulting in increased fatty acid concentrations in the blood and their subsequent uptake and storage in non-adipose tissues such as the liver and the skeletal muscle [32]. Saturated fatty acids, as well as di- and triglycerides exert their lipotoxic effects through inducing insulin resistance in various tissues; they also cause oxidative and endoplasmatic reticulum stress, the latter resulting in further stimulation of lipolysis [31]. Saturated fatty acids are also thought to induce the production of pro-inflammatory cytokines, leading to a state of low-grade, chronic inflammation in individuals with OWO [31]. Hence, excess body fat induces a spiral of negative consequences in lipid and glucose metabolism that perpetuates itself. Lipotoxic effects have been demonstrated also in the placenta of women with OWO [33].

## Lipid metabolism in normal pregnancy and in women with pre-pregnancy overweight, obesity and excessive gestational weight gain

### Pregnancy metabolism is characterized by hyperlipidemia

**Fig. 4:**
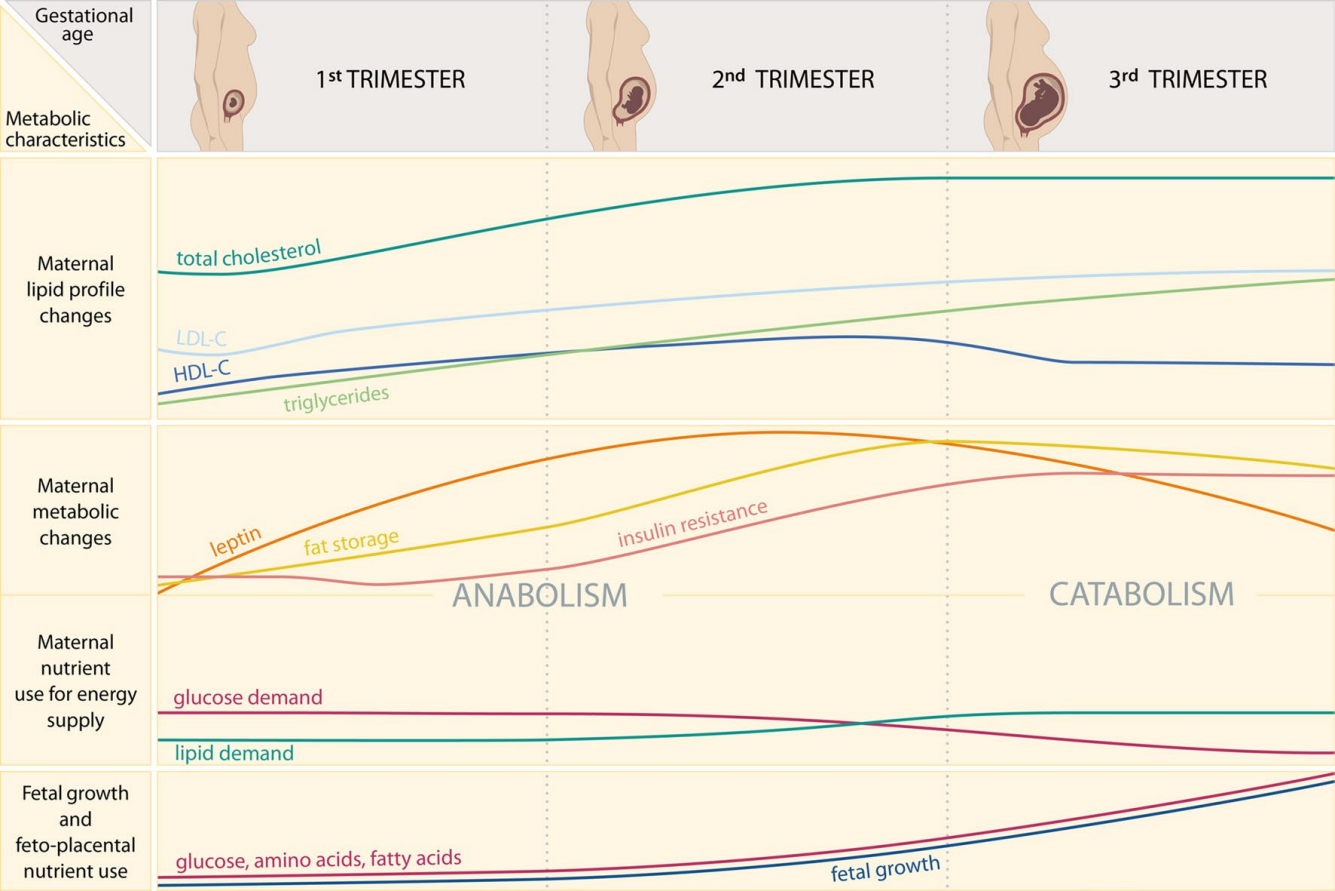
Standard lipid profile changes and metabolic adaptations during healthy pregnancy. Top: Maternal hyperlipidemia develops physiologically with increasing gestational age and is most pronounced in the third trimester, when fetal growth is accelerated. Triglycerides, total cholesterol, HDL-C and LDL-C are elevated in pregnant women [36] as compared to non-pregnant women [37–41], with variations in women of different ethnicities [42]. The increase of HDL-C occurs earlier than the increase of LDL-C [43], though the HDL-C concentration slightly drops again in the 3rd trimester [37, 39, 44]. Middle: Maternal metabolic changes throughout pregnancy are divided into two phases: during the anabolic phase in the first and second trimester, maternal fat storage is promoted [45–48] and leptin levels increase [49, 50], along with a slight increase in [51, 52] or unchanged [53] insulin sensitivity. Noteworthy, maternal fasting plasma glucose slightly dips in the first trimester [51, 54]. In the third trimester, maternal metabolism switches to a catabolic state, which is characterized by increased insulin resistance (starting already in mid-pregnancy) [51, 55] and increased lipolysis [56]. This is thought to benefit the supply of glucose and amino acids to the feto-placental unit to meet the nutritional needs in the phase of accelerated fetal growth (bottom) [57, 58]. Within this concept, in order to spare key nutrients for the fetus, the maternal metabolism shifts more towards the use of lipids to cover her own energy needs [46, 56, 59, 60]. Increased adipose tissue lipolysis may contribute to a smaller increase or stagnation of maternal fat mass accumulation in the third trimester [45, 48], however other reports have shown a linear increase of fat mass throughout the whole gestation [47]. Overall, longitudinal and reliable assessments of body fat are scarce. The trajectories shown for the respective parameters represent estimated changes throughout pregnancy using multiple of the abovementioned references and are not to be interpreted as absolute values or proportions. The trajectory for maternal insulin resistance was combined from multiple references reporting either insulin resistance, insulin sensitivity, or insulin secretory response in pregnancy. HDL-C: cholesterol within high density lipoprotein; LDL-C: cholesterol within low density lipoprotein. Images of pregnant women are copyrighted by BioRender

The maternal metabolism undergoes several physiological changes throughout normally progressing pregnancy (reviewed extensively in references [34, 35] and outlined in Fig. 4). Triglycerides, total cholesterol, HDL-C and LDL-C are elevated in pregnant women [36] as compared to non-pregnant women [37–41], with variations in women of different ethnicities [42].

In the context of maternal OWO, lipid levels can exceed the physiological range [61] and women with pre-pregnancy OWO were shown to have significantly higher concentrations of triglycerides (which was also observed in women with higher GWG [44]), free fatty acids, total cholesterol and LDL-C as well as lower concentrations of HDL-C compared to women with a normal pre-pregnancy BMI [39, 62–66]. Within HDL, cholesterol is transported from the periphery to the liver, where it can be converted into bile acids. A fraction of these bile acids are subsequently eliminated, providing the main mechanism to withdraw cholesterol from the circulation [28], next to transintestinal cholesterol excretion [67]. Hence, lower HDL-C levels and subsequent cholesterol accumulation are associated with atherosclerosis and metabolic syndrome [68]. A study investigating HDL functionality in pregnancy reported lower HDL-cholesterol, an increase in HDL cholesterol efflux capacity as well as a decrease in lecithin-cholesteryl acyltransferase (LCAT, an enzyme important for HDL maturation) activity in mothers with obesity with and without GDM as compared to pregnant women with normal weight [69]. These results were largely mirrored in the neonates, and, albeit not reaching statistical significance, may indicate an influence of maternal pre-pregnancy BMI on maternal and fetal lipoprotein metabolism [69]. Furthermore, early pregnancy levels of ApoB, and the ApoB/ApoA ratio were found to be positively associated with pre-pregnancy BMI [66], which is consistent with the atherogenic dyslipidemia phenotype often found in non-pregnant individuals with OWO.

To differentiate between physiological hyperlipidemia and pathological dyslipidemia in pregnancy, reference values for the standard lipid profile have been suggested in the 1990s [37] and since have been updated [36, 70–72]. Both, levels below or above the recommended ranges, can result in negative perinatal outcomes [36]. Thus, it would be advisable to include a lipid profile assessment in routine pregnancy care as a cost-effective method that allows to estimate potential risks for mother and fetus. Such lipid assessment is yet excluded from routine care, at least in Germany.

### Fatty acids are elevated in pregnancies complicated by overweight and obesity

As mentioned above, free fatty acids are associated with pre-pregnancy BMI. Hellmuth et al. demonstrated this association by reporting increased levels of non-esterified saturated and monounsaturated fatty acids, as well as elevated ratios of palmitoleic to palmitic (16:1 vs. 16:0) and oleic to stearic (18:1 vs. 18:0) acids with higher pre-pregnancy BMI [65]. The authors suggest this to be a consequence of stearoyl-CoA-desaturase (SCD-1) upregulation and subsequent promotion of lipid accumulation in liver and skeletal muscle in OWO, possibly leading to insulin resistance [65]. Indeed, SCD1 converts saturated into monounsaturated fatty acids, and enhanced SCD-1 activity and the resulting higher amounts of monounsaturated fatty acids have been associated with obesity and insulin resistance [73, 74]. Interestingly, SCD-1 is regulated by the diet, and SCD-1 deficiency in a mouse model of multiple sclerosis has recently been shown to promote regulatory T cell differentiation [75]. Transferring these findings to pregnancy, it is interesting to speculate that higher SCD-1 activity in pregnant women with OWO (as suggested, e.g., by higher SCD-1 protein concentration [76]) may (1) promote insulin resistance and (2) impair the induction and maintenance of the tolerogenic environment needed for fetal accommodation [77] and may thus be related to, e.g., the observed higher rate of abortion in women with OWO. However, these are highly speculative hypotheses which require further research and reliable confirmation.

Similar findings from the Generation R study revealed an association between excessive or higher GWG and higher mid-gestational levels of saturated, monounsaturated, and polyunsaturated (see below) fatty acids, independent of maternal pre-pregnancy BMI [64]. These observations may originate from a general increase in lipid levels in women with higher GWG. To evaluate the biological relevance of specific lipid alterations, e.g., in disease pathogenesis, it may be beneficial to consider the lipid composition (rather than the absolute concentration), which indicates the relative contribution of a specific lipid to the total lipid pool in a sample. Using the lipid composition, disruptions of the physiological ratios of lipids to one another can be displayed, which may be more informative than absolute concentrations in the comparison of disease states.

### Pro-inflammatory omega-6 polyunsaturated fatty acids are elevated in pregnant women with overweight and obesity

The essential polyunsaturated fatty acids (PUFA; Fig. 3) α-linolenic acid (18:3n-3) and linoleic acid (18:2n-6), as well as their respective derivatives docosahexaenoic acid (DHA, 22:6n-3) and arachidonic acid (20:4n-6) are particularly relevant during pregnancy, as they are required for fetal brain and retina development [78, 79]. Fatty acids are vertically transported from mother to fetus via placental lipid transporters with a preference for PUFA species [34, 80], which is reflected in their selective enrichment in the fetal circulation in a process termed biomagnification [61]. An elevation of DHA- and arachidonic acid-containing phospholipids was shown during pregnancy [81], and non-esterified PUFA decrease throughout pregnancy, which may result from a direct placental uptake or esterification to other lipid species [82].

Pre-pregnancy BMI has been shown to be positively associated with the amount of both free fatty acids and fatty acids within glycerophospholipids throughout gestation [64–66]. Specifically, the n-6 PUFAs dihomo-gamma-linoleic acid (DGLA; 20:3n-6), arachidonic acid, and adrenic acid (22:4n-6) were found to be higher, while linoleic acid was found to be reduced in women with higher pre-pregnancy BMI [64, 65]. On the other hand, the mid-gestational levels of the n-3 PUFAs α-linolenic and eicosapentaenoic acid (EPA; 20:5n-3) were lower in women with pre-pregnancy OWO than in pregnant women with normal weight [64]. Within plasma glycerophospholipids of women in mid-pregnancy with excessive or higher GWG, higher n-6 and n-3 PUFA, and a trend towards a higher n-6 to n-3 PUFA ratio was observed independently of maternal pre-pregnancy BMI [64]. In line with these observations, after delivery, a higher n-6 to n-3 PUFA ratio was observed in women with pre-pregnancy OWO [83]. Nowadays, Western diet (high in fat and sugar, low in fiber) habits are increasingly common and associated with higher dietary n-6 PUFA and lower n-3 PUFA content [84]. A higher dietary n-6 to n-3 PUFA ratio is linked to OWO, inflammation and increased risk for civilizational illnesses, whereas a lower ratio, resulting from higher n3-PUFA levels which can be obtained, e.g., by eating fish, is considered to have health benefits [84]. It has been proposed that the overall balance of n-3 and n-6 PUFA is essential to maintain a healthy equilibrium between pro- and anti-inflammatory effects, a suggestion which complicates the interpretation of PUFA analysis in the context of pregnancy. The observation of higher n-6 PUFA levels in women with higher GWG or OWO may support the notion that these conditions are associated with low-grade systemic inflammation. However, insights on lipidome changes that characterize gestational weight gain categories are still ambiguous, as another study failed to observe an association between GWG and plasma lipid profile [65]. This ambiguity may originate from the challenge to disentangle lipid metabolism changes that are triggered by excessive GWG vs. by higher pre-pregnancy BMI, as these conditions often co-occur. Interestingly, in the aforementioned studies, no associations were found between gestational lipid concentrations and the maternal dietary quality or quantity [65] or total energy and fat intake [64]. These dietary records did not assess detailed dietary PUFA content, which may be a reason for the lack of association with plasma PUFA concentrations. However, these findings could also indicate that the OWO-related alteration of fatty acid concentrations may originate from lipids stored in adipocytes that are brought into circulation again via lipolysis, or might result from the disturbance of the PUFA biomagnification process in pregnant women with OWO [61]. The latter is indicated by a lower content of DHA and arachidonic acid in placentas of obese women compared to placentas of women with normal weight [85]. In turn, this may explain the higher maternal PUFA levels in women with OWO compared to pregnant women who had normal weight prior to pregnancy. Given that n-6 PUFA tended to be selectively elevated, the proposed dysregulation in placental PUFA transport may also be specific to n-6 PUFA or involve enhanced n-3 PUFA over n-6 PUFA transport.

### Phospholipids increase during pregnancy and sphingolipids are linked to impaired glucose tolerance

When comparing the lipidomes of pregnant women in their second trimester to those 4–5 years postpartum, Mir et al. observed markedly higher phospholipids in pregnancy, most pronounced in phosphatidylethanolamines [81]. Interestingly, also the ratio of phosphatidylcholines to phosphatidylethanolamines was found to be lower in pregnancy, indicating a compositional change of the phospholipid pool during pregnancy [81]. Further, lower concentrations or decreasing levels of several lyso-phospholipids were found in pregnancy [82, 86, 87]. This decrease, especially in PUFA-carrying lysophospholipids [81] may be a reflection of their placental uptake to support fetal PUFA demand. Of note, complex lipids cannot cross the placenta, thus, placental lipases such as lipoprotein lipase and placental endothelial lipase are needed to release fatty acids from triglycerides and phospholipids [56]. Results from animal models indicate that placental lipoprotein lipase gene expression and activity are positively linked with increased neonatal adiposity in offspring of mice fed a high fat diet during gestation [88]. This strengthens the notion that placental lipases may increase the amount of fatty acids available for materno-fetal transport and hence, might be interesting targets in the context of maternal OWO.

In women with OWO, the ratio of total diacyl-phosphatidylcholines to acyl-alkyl-phosphatidylcholines were significantly elevated in the first trimester of pregnancy as compared to women with normal- or underweight [62]. Also, in blood samples from women with OWO taken between gestational week 15 and 35, a positive association between phosphatidylinositol (16:0_16:1), phosphatidylcholine (16:0_20:4) and (16:0_22:5) and GWG was identified [89]. Further, seven ether-phosphatidylcholines, most of which carried arachidonic acid and some palmitic acid (16:0) as side chains, two polyunsaturated phosphatidylcholines and several other lipids were negatively associated with GWG [89]. These findings highlight that different fatty acid compositions within the same lipid family (e.g., within phosphatidylcholines) or the same fatty acids bound to lipids with just slight structural differences (e.g., palmitic acid bound to either phosphatidylcholine or ether-phosphatidylcholine) may have opposing effects. In addition, some of the reported saturated and monounsaturated fatty acids associated with excessive GWG and OWO are products of de novo lipogenesis [64, 89]. De novo lipogenesis occurs in normal pregnancy, as shown in animal models [90], and could also be suspected as the origin of elevated triglycerides (e.g., TG 50:1, 52:2) detected in pregnancy by Rico et al. [91]. In excessive GWG, de novo lipogenesis may be enhanced, contributing to higher fat accumulation. However, this can only be speculated since the origin of the fatty acids has not been determined and hence is unknown.

Regarding sphingolipid metabolites, normal pregnancy was associated with increased levels of sphingomyelins [82, 86, 87] and ceramides (total, and, e.g., 16:1, 18:0, 20:0, 24:1) [86, 91], however also lower levels of ceramide (d19:1/24:0) [81] and lactosyl-ceramide (18:0) were observed in pregnancy [91]. Apart from being building blocks of cell membranes, sphingomyelins and ceramides act as signaling molecules in various cellular processes, such as migration, inflammation, apoptosis, and signal transduction [92]. Temporal clustering of lipid classes throughout pregnancy revealed that sphingomyelin and ceramide concentrations tend to be lower in the second trimester of pregnancy [86], which coincides with a period where enhanced immunotolerance and lower inflammation in the mother are needed to maintain the pregnancy [77]. A higher pre-pregnancy BMI and maternal pre-pregnancy OWO have been demonstrated to be positively associated with the amount of sphingomyelins with two double bonds (e.g., (34:2), (36:2), (36:3), and others), in early pregnancy [62, 65]. Further, data acquired within a small, but longitudinal study support that plasma ceramides are significantly reduced in mothers with OWO at birth, as compared to mothers with a normal BMI [93]. The participants of these studies were non-diabetic [65] or had low blood glucose levels in early pregnancy [62]. Nonetheless, women affected by pre-pregnancy OWO may enter pregnancy already in a state of low-grade insulin resistance, which is yet masked by euglycemia, but can lower the threshold to develop GDM [94]. In mice, sphingomyelins and ceramides have been associated with impaired glucose tolerance and insulin resistance [95, 96], and accumulation of ceramides in skeletal muscle and adipocytes in vitro has been shown to impair insulin signaling, resulting in reduced insulin sensitivity [97, 98]. Sphingomyelins are a major component of so-called “lipid rafts”, which are membrane domains that are important in insulin signaling [95]. In mouse models, it has been shown that genetic knockout [95] or pharmacological inhibition [96] of the enzymes that facilitate sphingomyelin biosynthesis lead to increased insulin sensitivity. Transferring these observations to pregnancy, it is tempting to speculate that sphingolipid metabolites may influence physiological and pathological insulin resistance throughout pregnancy.

Taken together, the presented studies indicate that higher maternal pre-pregnancy BMI and maternal OWO are associated with higher levels of triglycerides, total cholesterol, LDL-C, free fatty acids, saturated fatty acids, n-6 PUFA, sphingomyelins and phosphatidylcholines, whereas levels of HDL-C, n-3 PUFA and ceramides seem to be lower in these women as compared to women with normal pre-pregnancy BMI. Clearly, the interdependencies between sphingolipid metabolism, OWO and insulin resistance during pregnancy should be addressed in future research endeavors.

## Lipid alterations in the development of gestational diabetes mellitus and hypertensive disorders during pregnancy

Lipidomic studies hold the potential to identify lipid biomarkers indicative for adverse health outcomes in mother and child. As recently shown, the prediction of GDM, HDP and LGA neonates yielded a high accuracy when including novel lipid biomarkers in the prediction models [99–103]. Of note, the studies discussed below included women both with and without pre-pregnancy OWO; OWO-specific results were emphasized where applicable.

It is well known that the risk for GDM, a common pregnancy complication defined by the occurrence of hyperglycemia at any time during pregnancy [104], is increased in women with a higher BMI and higher age, along with other parameters [94]. It has also been suggested that women carrying a male fetus may be at a slightly higher risk for GDM, which could be a consequence of pancreatic β-cell impairment [105, 106]. The current prevalence of GDM amounts up to 30% of pregnancies [94], and will likely further increase due to the rise of OWO prevalence and nowadays higher maternal age at delivery. GDM predisposes for additional adverse pregnancy outcomes, such as HDP/pre-eclampsia, fetal macrosomia and LGA, preterm birth, as well as complications during labor and delivery. Children born to mothers with GDM are affected by the higher risk for neonatal complications, such as neonatal hypoglycemia [94]. Furthermore, mother and children alike are more prone to develop type II diabetes mellitus [94, 107] and cardiovascular diseases later in life [108]. Despite the fact that the association between glucose and lipid metabolism in pregnancy is well-known, the specific influence of maternal lipids on the risk of GDM development is not yet fully understood. The assessment of standard lipid profiles in GDM-affected pregnancies revealed a positive association between triglycerides and total cholesterol with GDM risk, whilst HDL-C was inversely correlated with the GDM risk [109–111]. Triglyceride levels seemed to have the strongest effect on the GDM risk, particularly during the last third of pregnancy [111]. Of note, in the pathogenesis of type II diabetes mellitus, insulin resistance leads to an increase of hepatic triglyceride synthesis and their subsequent release into the circulation [32]. Applying this concept to GDM, it is unsure whether the observed elevated triglyceride concentrations truly are a risk factor for the development of GDM, or if they represent a marker of the already developed insulin resistance. The Triglyceride/Glucose (TyG) index may also serve as a surrogate marker for insulin resistance in this respect [112]. Additionally, GDM-affected women show a higher ApoB/ApoA1-ratio [113], which suggests that atherogenic lipid profile changes may be related to the observed long-term cardiovascular health sequelae in women affected by GDM.

In addition to GDM, pre-eclampsia is also a severe, multi-system pregnancy complication affecting approximately 2–5% of pregnancies [114]. Among other, maternal obesity, an advanced maternal age (≥ 35 years at delivery), nulliparity and ethnicity (e.g., Afro-Caribbean, South Asian) are recognized risk factors for pre-eclampsia [114, 115]. Clinical symptoms generally commence from gestational week 20 and include gestational hypertension, accompanied by organ manifestation such as proteinuria or uteroplacental dysfunction [114]. Pre-eclampsia is subdivided into the four subtypes of early- and late onset (delivery < 34 + 0, or ≥ 34 + 0 gestational weeks, respectively), as well as preterm- and term pre-eclampsia (delivery < 37, or ≥ 37 + 0 gestational weeks, respectively) [114]. Pre-eclampsia and gestational hypertension are summarized as HDP, which are a leading cause for perinatal mortality, causing up to 26% of maternal deaths depending on geographic location [116]. Pre-eclampsia also has long-term health sequelae for mother and child after birth, as it increases the risk for cardiovascular and metabolic diseases in both [117]. The development of pre-eclampsia is not fully understood, but is considered to result from a two-stage paradigm: poor placental development in early pregnancy promotes placental ischemia and oxidative stress, which subsequently leads to systemic endothelial dysfunction in the mother which underlies the clinical symptoms [115, 117]. Notably, women carrying a male fetus seem to be at a slightly higher risk for term pre-eclampsia than those carrying a female fetus, mainly in non-Asian populations [105, 118], while pre-term pre-eclampsia was repeatedly associated with female fetal sex [105, 119, 120]. This sex-dimorphic effect has been theorized to result from a higher susceptibility of male fetuses to impaired implantation and placentation, which would result in more spontaneous miscarriages (and subsequently less male fetuses whose mothers can be diagnosed with preterm pre-eclampsia) than in females [105, 119, 121]; furthermore, heightened placental inflammatory markers were found in term pre-eclampsia in placentas of male as opposed to placentas of female [122].

Dyslipidemia has been linked to the pathogenesis of pre-eclampsia [114], but respective pathways are still largely unknown or obscured by ambiguous observation. Individual studies as well as a large meta-analysis assessing standard lipid profiles in the context of HDP/pre-eclampsia revealed elevated levels of total cholesterol, triglycerides, VLDL, ApoB, ApoA1, Lp(a), atherogenic lipid indices and non-HDL-C in women with HDP/pre-eclampsia, whereas HDL-C tended to be decreased in pre-eclamptic women during the third trimester of pregnancy [41, 44, 111, 123–127]. GDM is a well-known risk factor for the development of HDP/pre-eclampsia and lipid profile alterations, specifically in triglycerides, may underlie this co-morbidity [41, 44, 125]. These findings further underscore that GDM and HDP/pre-eclampsia are closely linked, sharing not only predisposing factors and potential complications, but also highly similar changes in lipid profiles.

### Gestational diabetes is accompanied by increases in de novo lipogenesis-related fatty acid species

A wealth of individual lipid metabolites has been shown to be altered in GDM or HPD/pre-eclampsia development. To date, there is large heterogeneity within lipidomic studies that investigate the influence of maternal lipid markers on pregnancy complications. Studies lack comparability due to varying methodology in lipid extraction and measuring devices, targeted vs. untargeted lipidomics approaches, use of fasted vs. non-fasted and plasma vs. serum samples, composite endpoints vs. single entities as outcomes, differing cohort sizes and study design (cross-sectional vs. longitudinal, retrospective vs. prospective), selection of participants (inclusion and exclusion criteria), blood sampling timepoints (gestational age ranges), non-diverse ethnicity, differences in statistical analysis and adjustment for confounders as well as in the nomenclature of detected lipids and the reporting of results.

**Fig. 5:**
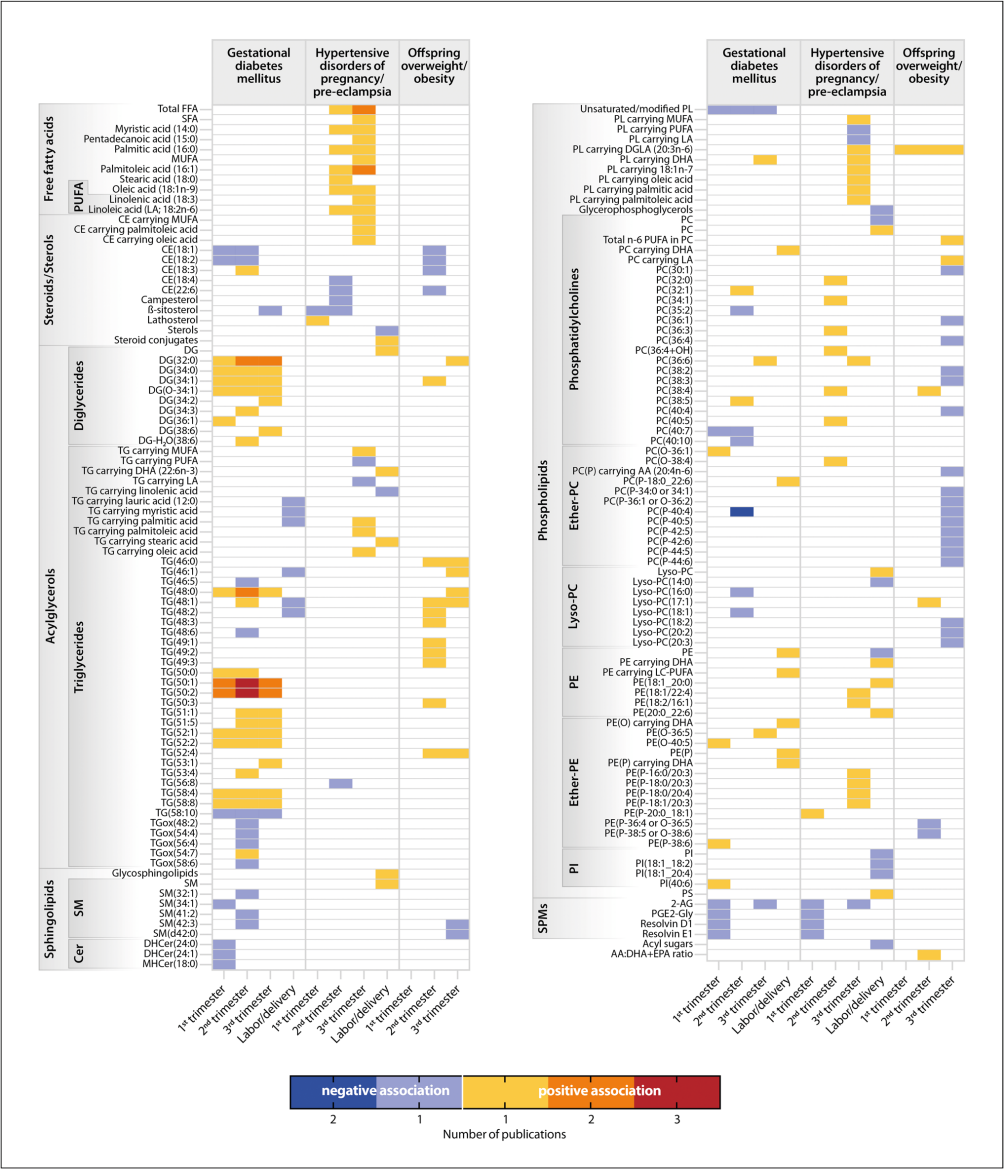
Maternal blood lipidome in association with gestational diabetes mellitus, hypertensive disorders of pregnancy/pre-eclampsia and offspring overweight and obesity. The heatmap visualizes data reported in references [86, 99, 101, 103, 129–134] for GDM, in references [99, 102, 123, 128, 132, 135–137] for HDP/pre-eclampsia and in references [129, 138–142] for offspring overweight and obesity. As indicators of offspring overweight and obesity, the following were considered: birthweight, birthweight z-score and percentile, LGA, abdominal circumference, percentage of body fat, and skinfold thickness. The color code carries double information: Yellow, orange and red each represent a positive association with/higher amounts of the respective lipid in the observed condition, while light blue and dark blue represent a negative association with/lower values of the respective lipid in the observed condition. The color code indicates the number of publications that the association was reported in, not the strength of the association. White boxes represent missing data. E.g.: In three studies, a positive association (red box) between the amount of triglyceride (50:1) in the second trimester of pregnancy and the development of gestational diabetes mellitus was found. In one study, a negative association (light blue box) between cholesteryl ester (18:1) in the second trimester of pregnancy and the development of offspring overweight and obesity was found. “PC” has been entered twice, since two studies found opposing results. The large number of white boxes indicates the heterogeneity of studies. Presented studies included women with and without pre-pregnancy OWO, and in the case of studies regarding offspring adiposity also women with and without GDM. If the gestational age at study visit overlapped in trimester affiliation, the findings were either placed in the trimester that was most fitting, or in both trimesters based on the assessed gestational age ranges. To facilitate the comparison of findings, individual fatty acid chains were summed up for DG and TG species. Blood lipidome refers to plasma and serum samples. A reference-annotated version of the heatmap with information about the included studies can be found in the Supplementary material. AA: arachidonic acid; CE: cholesterol ester; Cer: ceramide; DG: diglyceride; DGLA: dihomo-gamma-linoleic acid; DHA: docosahexaenoic acid; DHCer: dihexosyl ceramide; EPA: eicosapentaenoic acid; LA: linoleic acid; Lyso-PC: lysophosphatidylcholine; MHCer: monohexosyl ceramide; MUFA: monounsaturated fatty acids; n-6 PUFA: omega-6 polyunsaturated fatty acids; PC: phosphatidylcholine; PC(O-): alkyl ether phosphatidylcholine; PC(P-): phosphatidylcholine plasmalogen; PE: phosphatidylethanolamine; PE(O-): alkyl ether phosphatidylethanolamine; PE(P-): phosphatidylethanolamine plasmalogen; PGE2-Gly: prostaglandin E2-glyceryl ester; PI: phosphatidylinositol; PL: phospholipids; PS: phosphatidylserine; PUFA: polyunsaturated fatty acids; SFA: saturated fatty acids; SM: sphingomyelin; SPMs: specialized pro-resolving mediators; TG: triglyceride; TGox: oxidized triglyceride; 2-AG: 2-arachidonoylglycerol

In order to shed some light into the currently available insights, but also to highlight the gaps, ambiguity and heterogeneity of the evidence published to date, we developed a structured overview of lipids identified to be associated with the development of GDM or HDP/pre-eclampsia (Fig. 5) [86, 99, 101–103, 123, 128–137]. This overview also integrates findings from the UK-based UPBEAT trial, which revealed that women with obesity who later developed GDM presented higher concentrations of di- and triglycerides containing palmitic, palmitoleic, stearic and oleic acid than women with obesity who did not develop GDM [129, 131], findings that were replicated also in other cohorts [86]. As described above, these fatty acids may be products of hepatic de novo lipogenesis and thus, it is conceivable that a higher rate of de novo lipogenesis could be involved in the pathogenesis of GDM. Interestingly, these elevated di- and triglycerides were not associated with maternal BMI, but influenced by maternal ethnicity [86], magnitude of maternal hyperglycemia and insulin resistance [129]. Again, in the association of these lipid species with GDM, cause and consequence are indistinct (see above). However, the ability to detect specific lipid alterations before the routine GDM test may be helpful in making a timely diagnosis.

### Polyunsaturated fatty acids and their derivatives are dysregulated in gestational diabetes and pre-eclampsia

Overall, clustering of positive associations with GDM development can be seen in the di-and triglyceride classes, whereas in HDP/pre-eclampsia, an abundance of free fatty acids was found to be upregulated and thought to be the result of enhanced lipolysis due to oxidative stress and inflammation [123, 135] (Fig. 5). Regarding cholesterol metabolism, the levels of lipid markers indicating cholesterol absorption and synthesis in women who developed GDM [134] or pre-eclampsia [128] were suggested to represent disturbances in the cholesterol homeostasis, which may be involved in the pathogenesis of GDM and pre-eclampsia. DHA-containing phospholipids and specialized pro-resolving mediators (SPMs) have also been tested in the context of cardiometabolic complications (GDM and HDP as combined endpoint) in pregnancy [99]. SPMs are a group of lipid mediators derived from PUFA that are considered to resolve inflammation [99]. In women with cardiometabolic complications, the amount of DHA in the phospholipid fraction was significantly higher in the third trimester as compared to women with uncomplicated pregnancies [99]. This elevation was interpreted to be driven by PC(36:6), which carried the fatty acids myristic acid and DHA [99]. Among the SPMs, in the first trimester, the DHA- and EPA-derived [21] resolvins D1, D2, and E1 as well as the arachidonic acid-derived 2-arachidonoylglycerol (2-AG) and prostaglandin E2-glyceryl ester were significantly lower in the serum of women with cardiometabolic complications, while in later pregnancy, only 2-AG remained lower in those women [99]. These findings may lead to the conclusion that GDM and HDP/pre-eclampsia are promoted by a lack of SPMs and a subsequent accumulation of DHA, which could be due to impaired DHA biomagnification or an enzymatic insufficiency to transform DHA into its downstream anti-inflammatory mediators such as SPMs and anti-inflammatory eicosanoids, thus leading to increased pro-inflammatory stimuli. However, these assumptions are somewhat contradictory, as DHA also exerts direct anti-inflammatory properties via the G-protein coupled receptor (GPR) 120 in various tissues [143] and thus, one could expect more anti-inflammatory effects if DHA levels are higher. Nonetheless, elevated systemic inflammation is seen as a consequence of OWO-associated lipotoxicity [31] and is a condition shared with pre-eclampsia [117] and GDM [94]. It thus likely contributes to the pathogenesis of these diseases, but the role of PUFA remains to be elucidated.

Taken together, lipid profile and lipidomic analysis prior to the onset of GDM or HDP/pre-eclampsia may help to advance an early detection of these severe pregnancy complications, although there is still ambiguity in study results. In the case of GDM, such early detection would increase the time period for treatment of GDM during gestation via adequate education including, e.g., diet plans, exercise recommendations or insulin treatment, hereby improving maternal and fetal metabolism as well as future health. In regards to HDP/pre-eclampsia, a reliable lipid biomarker screening may, once established after more in-depth confirmatory studies, facilitate early preventive treatment, e.g., with aspirin [117] or statins within clinical trials [144] and intensified prenatal observation.

## Maternal lipids and offspring’s body composition

It has long been observed that children of mothers with OWO are more often OWO themselves [145], and rising OWO prevalence in children has been observed globally, paralleling the same trend as in adults [146]. Anthropometrics in neonates, infants and children are – next to (birth)weight, height, and BMI – based on skinfold thickness, abdominal and waist circumference, waist-to-height-ratio and body composition and can indicate offspring OWO. To evaluate these indices, stratification based on the child’s age, sex and ethnicity is required and thus, findings are generally compared using age- and sex-adjusted, region-specific percentile curves or z-scores. As for the assessment of fetal weight gain, ultrasound is the method of choice to, e.g., detect fetal macrosomia (birthweight greater than 4000 g) [147]. In fact, neonatal morbidity and infant mortality increase in a dose-dependent manner if the birthweight is greater than 4500–5000 g, respectively [148].

In a number of studies, maternal OWO and -related lipid dysregulation were linked with childhood OWO. A possible explanation lies in the fetal oversupply of fatty acids and glucose that results from maternal overnutrition in OWO pregnancy and may prime for later obesity [149]. Large meta-analysis of more than 150,000 mother-offspring pairs as well as independent studies described an increasing risk for offspring OWO with higher maternal pre-pregnancy BMI and excessive GWG, independently of maternal GDM and glucose levels [150, 151]. Strikingly, in a mediation analysis, the effect of fasting plasma glucose on macrosomia was fully mediated by ApoB and the TG/HDL-ratio in a Chinese cohort [152], which highlights the interplay of maternal atherogenic lipoprotein levels and glucose metabolism in the development of neonatal adiposity. Lipidomic studies in this field (examples found in Fig. 5 [129, 138–142]), are heterogenous in many aspects, as mentioned above. In the following, we would like to highlight some of the potential lipids and pathways involved.

### Maternal triglycerides are linked to indicators of newborn adiposity

Maternal lipid levels in early to mid-pregnancy, such as higher triglycerides, total cholesterol and LDL-C, were positively associated with birthweight as well as LGA and fetal macrosomia, whilst HDL-C was negatively associated with birthweight, and linked to an increased risk for SGA [40, 111, 150, 153–155]. These results indicate that the maternal standard lipid profile can, in addition to ultrasound-based fetal weight estimation, aid in the prediction of birthweight and birthweight-related outcomes, and could be utilized to predict, e.g., macrosomia [152]. Specific maternal triglycerides, e.g., 46:0 and 48:1, that were positively associated with indicators of newborn adiposity, may originate from de novo lipogenesis [129, 139]. Complex network analysis revealed that also the longitudinal trajectories of specific lipids throughout pregnancy were associated with neonatal anthropometry [139]. Adding specific lipid species (amongst other metabolites) to a prediction model for birthweight that was based on maternal pre-pregnancy BMI increased the percentage of variance explained by the model as compared to prediction by conventional biomarkers [62]. Further, Hellmuth et al. suggested a protective effect of maternal third trimester ether-phosphatidylcholines carrying arachidonic acid on neonatal body fat percentage [140]. This would, vice versa, imply that lower levels of these lipids in the maternal circulation would be associated with an increase in fetal body fat accumulation. As a theoretic concept, this could be explained by an increased liberation of arachidonic acid from maternal phospholipids within the placenta, resulting in higher transport rates of arachidonic acid to the fetus. Such increased liberation may be caused by overexpression of the placental lipases, or of placental fatty acid transporters that either favor arachidonic acid transportation or increase fatty acid transport overall. Increased levels of arachidonic acid in the fetus may prime for childhood OWO by favoring a pro-inflammatory milieu; however, the specific mechanisms linking (maternal obesity-induced) inflammation to offspring obesity are yet unknown.

### Maternal lipids and maternal overweight and obesity may prime offspring’s birthweight in a sexually dimorphic way

In most of these studies, the effect of maternal lipids on the offspring’s risk for OWO was not reported to be influenced by the child’s sex. Interestingly however, LaBarre et al. demonstrated sexually dimorphic effects of several maternal lipid groups in the first trimester and at term on the offspring’s birthweight z-score [156]. Independently of maternal lipids, Daraki et al. found an increased risk for childhood OWO and visceral adiposity at the age of 4 years in girls, but not boys, when born to women with pre-pregnancy OWO [63], and Sommer et al. reported a higher sum of skinfolds in female neonates as opposed to males [150]. A possible explanation for this may lie in a hypothesis posed by Eriksson and colleagues, who suggest that girls respond more to the general nutritional status of the mother, and direct their resources towards fat accumulation and placental growth, whereas boys tend to grow faster, making them more susceptible to nutrient restriction [157]. The prevalence of OWO in childhood has consistently been shown to be higher in boys than girls in various countries, which likely results from a combination of biological sex-based, and sociocultural gender-based influences [158]. However, the role of maternal lipids in this context needs to be further elucidated.

### Lipids linked to pro-inflammatory stimuli and oxidative stress may play a role in the priming of childhood obesity

For the prediction of OWO in childhood, higher maternal levels of triglycerides, total cholesterol, ApoB and free fatty acids during pregnancy were positively associated with offspring OWO at 3 to 12 years of age [63, 66, 113, 159, 160]. A lower increase of body weight until the age of 8 years was shown when maternal mid-gestational HDL-C and ApoA1 levels were higher [159]. Other studies reported a positive association between maternal n-6 fatty acids within plasma phospholipids and OWO in childhood [141, 142]. For example, the maternal concentration of DGLA within phospholipids throughout gestation was positively associated with offspring OWO at the age of seven years [141]. DGLA derives from linoleic acid and can be converted into arachidonic acid at a limited rate [161]. Both, DGLA and arachidonic acid, are precursors for eicosanoids, a group of tissue hormones which are involved both in initiation and resolution of inflammation [21] and encompass leukotrienes, prostaglandins, thromboxanes, lipoxins and resolvins [162]. DGLA is metabolized into series 1 prostaglandins, which are considered as anti-inflammatory mediators, while arachidonic acid is metabolized to series 2 prostaglandins, which have pro-inflammatory functions [21, 162]. However, the evaluation of study results regarding eicosanoids is challenging due to their pleiotropic functions [162]. For example, prostaglandin PGE_2_ (derived from arachidonic acid) leads to increased vascular permeability and induces pro-inflammatory Th17 cells, while also decreasing differentiation and function of other pro-inflammatory T cell types [163]. Further, during the synthesis of prostaglandins, reactive oxygen species are released as a byproduct [161]. Hence, increased levels of maternal DGLA, if processed into prostaglandins at a high rate, may lead to increased oxidative stress in the maternal body during pregnancy, which may prime the offspring for OWO via yet unknown mechanisms. Oxidative stress is defined as a dysbalance between reactive oxygen species and antioxidants, which leads to enhanced oxidation of cellular components [164]. Interestingly, oxidative stress also leads to lipid (per)oxidation – mainly of PUFA, due to their chemical reactivity – which damages cell membranes and alters cell signaling and protein function. Oxidative stress is also linked to inflammatory processes [164], and both have been shown to be increased in placentas of women with OWO [33]. Intriguingly, markers of oxidative stress such as 8-iso-prostaglandin F2α (8-iso-PGF2α) are higher in individuals with OWO and associated with cardiovascular and metabolic diseases [165]. During pregnancy, higher urinary concentrations of 8-iso-PGF2α have been associated with lower birthweight and (very) rapid infant weight gain in a dose-dependent manner [166]. Catch up growth or rapid infant weight gain often, but not exclusively, occurs in infants with a low birth weight or born prematurely and is seen as a compensatory mechanism for poor nutrient supply in utero [167, 168]. This trajectory of offspring’s weight gain has been strongly linked to a higher risk for obesity later in life [167]. Further, enhanced lipid peroxidation was also reported in women with excessive GWG and shown to positively correlate with neonatal adiposity [169]. However, the sample size of these studies is still small and further research is needed to confirm the role of oxidative stress in offspring OWO development. Animal intervention studies targeting fetal metabolic programming in the context of maternal obesity have pointed to beneficial effects of dietary antioxidant supplementation, e.g., with resveratrol, on metabolic programming of the offspring; however, also toxic effects at high doses and effects on pancreatic development were observed (reviewed in [170, 171]). Importantly, corresponding evidence and safety assessments in human pregnancy are still lacking [171].

Notably, some studies showed only weak or even no associations between maternal lipid profile in pregnancy and offspring adiposity, suggesting alternative pathways of priming of offspring OWO [113, 154, 172, 173].

### Possible mechanisms of offspring metabolic priming include PPARγ signaling and epigenetic modifications

The development of fat cells in the fetus starts around gestational week 14–16, followed by fat cell proliferation until gestational week 23 [174]. Mechanisms of fetal adipose tissue development and fetal lipid metabolism are comprehensively reviewed in [175]. As fatty acids act as ligands for the transcription factor PPARγ, which is a key regulator of adipogenesis [176, 177], it can be speculated that fetal fatty acid oversupply originating from maternal overnutrition may lead to enhanced fetal PPARγ activation. Consequently, this may result in a higher number of adipocytes that can accumulate fat later in life and promote offspring OWO. Furthermore, excess supply of maternal lipids early in pregnancy, prior to fetal adipocyte differentiation, may result in ectopic fat accumulation, e.g., in the liver, subsequently increasing the offspring’s risk for metabolic dysfunction associated steatotic liver disease (MASLD; former: non-alcoholic fatty liver disease, NAFLD) [56, 178], as shown in nonhuman primate models [179]. Epigenetic modification of fetal genes is another potential mechanism by which maternal lipids may influence developmental programming and offspring’s health. As demonstrated in a metabolome study, pregnancy levels of very long chain fatty acids such as DHA were positively associated with methylation in genes known to be associated with metabolic health and disease [180]. Notably, it has also been suggested that the increased risk for offspring adiposity that results from higher maternal pre-pregnancy BMI is not mediated by maternal lipid levels [66]. As the majority of human studies in this field are of correlative rather than causal nature, the comprehension of mechanisms causally linking specific lipid species in the context of maternal OWO-related dyslipidemia to offspring OWO predisposition and metabolic complications later in life is still incomplete.

Animal models suggest metabolic advantages in offspring born to obese dams following dietary or exercise interventions before and during pregnancy as compared to no intervention [181, 182]. However, human lifestyle intervention studies targeting maternal OWO or excessive GWG mainly via dietary recommendations and exercise schedules mostly only start in pregnancy. They have so far demonstrated moderate to no effects in reduction of OWO in the offspring [183–186]. The use of glucagon-like peptide 1 agonists as a weight-management measure is not recommended in pregnancy due to potential adverse fetal outcomes [187]. For these reasons, it is a common understanding that lifestyle modifications including weight loss should ideally commence pre-conception in order to attenuate fetal programming through maternal OWO [188, 189].

Maternal excessive GWG was also associated with changes in the child’s lipid profile, such as higher triglyceride levels and lower HDL-C, even when adjusted for macronutrient intake and physical activity in the offspring [190]. This strongly underscores the long-term sequelae of maternal overnutrition during pregnancy on the offspring’s lipid metabolism. Pioneering work to strengthen this notion was provided in a study by León-Aguilar et al., who highlighted that specific lipidome changes seen in women with OWO at delivery were not detectable in cord blood, but emerged in the children at the age of 4 years [93]. This could be indicative for a long term “inheritance” of maternal lipid alterations that possibly link maternal OWO and its long-term influence on children’s health. However, the impact of gestational maternal lipid overload on offspring OWO risk seems to be attenuated in early adolescence [113]. A possible explanation may be that accelerated growth and thus, high energy expenditure in adolescence could temporarily mask the OWO predisposition which reemerges once adulthood is reached.

## Conclusions

Dysregulations in the lipid metabolism during pregnancy, which also occur in relation to maternal OWO, are associated with an increased risk for GDM and HDP, as well as with an increased birth weight and offspring’s risk for OWO later in life. Insights from studies available to date are still patchy and ambiguous due to the differences in design and variations or limitations in read out parameters. Elevated triglycerides, changes in phospholipid composition and a dysregulation in PUFA metabolism seem to be key modulators, but it is premature to convert insights available to date into clinical guidelines. Translational approaches using animal models, e.g., of diet- or genetically induced obesity, are widely recognized for the elucidation of pathogenetic pathways involved in OWO-associated diseases, and have found application also in models of fetal developmental programming in the context of maternal OWO, as well as in gestational diabetes (reviewed in [191–194]). However, there are still open questions regarding the transferability of animal lipidomes to the human condition. Plasma lipidomes of hamsters and mice are believed to be similar to human plasma lipidomes in a non-pregnant state [195], however large studies systematically investigating the comparability of lipidomes during human and animal pregnancy are largely missing (see Box 1). The advent of lipidomic profiling is promising to understand the mechanisms that drive pregnancy-related risks and health disadvantages in (OWO) mother and offspring and thus, more large-scale lipidomic studies and in-depth mechanistic research is needed. The resulting recommendations need to be transported into society in an understandable and easy-to-implement way in order to attempt bringing the vicious circle of OWO to a halt.


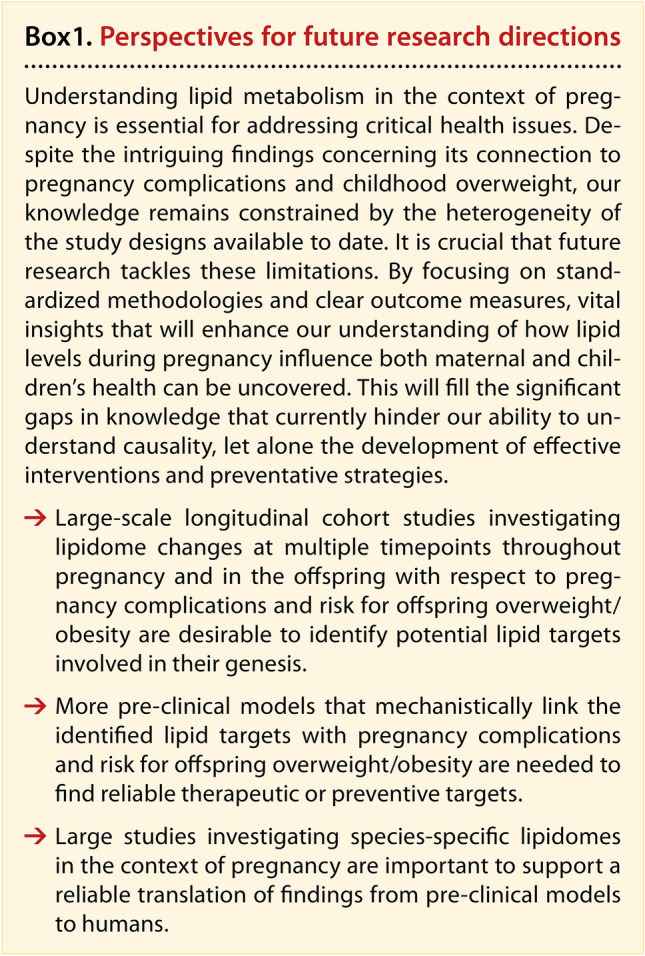


## Supplementary information

Below is the link to the electronic supplementary material.

ESM 1 (XLSX 27.8 KB)

## Data Availability

A reference-annotated version of the heatmap in Fig. 5 can be found in the Supplementary material.

## Footnotes

1 OWO is used throughout this article as an abbreviation for the following terms: overweight and/or obesity, overweight and/or obese.

## Declarations

### Illustrations

All figures were designed or modified using InDesign, Adobe Photoshop and Adobe Acrobat Pro. Images in Fig. 3 are copyrighted by BioRender. Figure 5 was created in GraphPad PRISM (version 10.2.3).
